# Effect of Aluminum Addition on the Microstructure and Properties of Non-Eutectic Sn-20Bi Solder Alloys

**DOI:** 10.3390/ma12071194

**Published:** 2019-04-11

**Authors:** Wenchao Yang, Jidong Li, Yitai Li, Junli Feng, Jingwu Wu, Xiankun Zhou, Aihua Yu, Jiahui Wang, Siyu Liang, Mei Wei, Yongzhong Zhan

**Affiliations:** 1 School of Resources, Environment and Materials, Guangxi University, Nanning 530004, China; ywc053@163.com (W.Y.); lijidong@163.com (J.L.); lyt8590@126.com (Y.L.); 18987231124@163.com (X.Z.); aihua828118@yeah.net (A.Y.); 13297967586@163.com (J.W.); lsyqgz0430@163.com (S.L.); sky1409010125@163.com (M.W.); 2 Guangxi Key Laboratory of Processing for Non-Ferrous Metals and Featured Materials, Nanning 530004, China; 3 School of Materials Science and Engineering, South China University of Technology, Guangzhou 510641, China; 4 Shenzhen Exit Inspection and Quarantine Bureau Industrial Products Inspection Technology Center, Shenzhen 518067, China; fjlhhp@foxmail.com (J.F.); wujingw@163.com (J.W.)

**Keywords:** non-eutectic, Sn-20Bi alloy, wettability, aluminum, lead-free

## Abstract

This study investigates the effect of aluminum (Al) on the microstructure, micro-hardness, and wettability of environmentally friendly Sn-20Bi-xAl (x = 0, 0.1, 0.3, 0.5 (wt.%)) solder alloys. Scanning electron microscopy (SEM) with energy dispersive spectroscopy (EDS) analysis, and X-ray diffraction (XRD), were used to identify the microstructure morphology and composition. The spreading area and contact angle of the Sn-20Bi-xAl alloys on Cu substrates were used to measure the wettability of solder alloys. The results indicate that Al increased the hardness to a maximum value of ~27.1 HV for x = 0.5. When the content of Al was more than 0.3 wt.%, the hardness change value gradually flattened. From the spreading test results, Al reduced the wettability of solder alloys. When the content of Al was 0.1 wt.%, the change was slight. When more than 0.3 wt.%, the wettability of Sn-20Bi-xAl solder alloys sharply dropped. The corrosion resistance of Sn-20Bi-0.1Al alloy was the best, and the corrosion rate was at the lowest value at 0.092 mm/a due to the dense corrosion products.

## 1. Introduction

Traditionally in the electronic packaging industry, conventional Sn-Pb solder has been a dominant material due to its good properties and low cost [1]. However, with increased concerns relating to human health and the environment, legislation and regulations have been established to eliminate or limit the usage of lead due to the toxic nature of Pb, such as the restriction of certain hazardous substances (RoHS), and waste electrical equipment (WEEE) [2]. Thus, the pursuit of alternative lead-free soldering materials has become inevitable.

Due to the development of lead-free soldering materials in recent decades, binary or ternary Sn-based alloys, including Sn-58Bi (138 °C) [3], Sn-9Zn (198 °C) [4], Sn-0.7Cu (227 °C) [5], Sn-3.5Ag (221 °C) [6], Sn-70Au (280 °C) [7], Sn-35Bi-1Ag (187 °C) [8] Sn-8Zn-3Bi (188 °C) [9], and Sn-3.0Ag-0.5Cu (217 °C) [10] systems, have been widely applied as replacements to Sn-38Pb (183 °C) systems, according to different packaging conditions. Nevertheless, the Sn-based lead-free alloys were found to not be suitable for traditional soldering equipment and processes, although the number of candidates was huge. Sn-Ag-Cu showed poorer creep and low wettability to copper surfaces. The cost of Ag was high too. Furthermore, their liquidus temperature (around 217 °C) was higher than Sn-37Pb at 183 °C [11]. The easy oxidation of Sn-Zn alloys was a fatal weakness. Eutectic Sn-58Bi solder was a significant solder alloy with good wettability, good mechanical properties, and a low melting point, which was only 138 °C [12]. The Sn-Bi solders have more potential for a low liquidus temperature. Many investigations about eutectic Sn-58Bi solder alloy have been conducted. However, due to the fragility and the poor ductility of eutectic Sn-58Bi solder alloy, it was not popularly used in the electronic packaging industry. Goh et al. reported that the Sn-58Bi eutectic alloy was hard and brittle because of its high content of Bi-rich phase [13]. Recently, non-eutectic Sn-xBi solder alloys have been the focus of much research due to superior properties compared with eutectic Sn-58Bi solder alloy. Shen et al. studied the mechanical properties of Sn-xBi (x = 3, 10, 50, 58, 70) alloys and found that Sn-xBi alloys with Bi concentrations up to 10 wt.% showed superior mechanical properties [14]. Li et al. discovered a novel Sn-20Bi solder by adding microelements (Ag, Cu, Ge, Ce, Sb) and decreasing the melting point to around 186 °C, which was close to the traditional eutectic Sn-37Pb solder alloy. Furthermore, mechanical and thermal properties of the novel solder were similar with those of the Sn-37Pb alloy [15]. Ye et al. investigated the microstructure, the mechanical properties, and the fracture behaviors of the non-eutectic Sn-Bi system. The morphology of the Bi phase was particle-shaped and became gradually irregular when the fraction of Bi increased, until up to 58% when the Bi phase became a continuous matrix. The microstructure of Sn-20Bi solder alloy did not lead to eutectic phase and consisted of Bi particle and β-Sn phase. The ultimate tensile load (UTL) of Sn-20Bi solder alloy was higher than that of Sn-10Bi and Sn-25Bi alloy [16,17]. Although the reduction of Bi content in Sn-xBi alloys was helpful for declining the fragility and increasing the ductility, the Bi concentration was diminishing the wettability of SnBi alloys on Cu substrate during soldering. The wettability of non-eutectic Sn-xBi solders diminished with decreasing Bi contents [18]. Furthermore, the mechanical properties of Sn-20Bi alloy were suppressed because of Bi segregation, and coarse Bi-rich Sn-20Bi alloys resulted from the large melting temperature range between solidus and liquidus.

Recently, several research groups have focused on manufacturing new alloys doped with micro-/nano-meter size particles, for example, nickel (Ni), iron (Fe), zinc (Zn), silver (Ag), aluminum (Al), bismuth (Bi), zirconia (ZrO_2_), alumina (A_l2_O_3_), titanium oxide (TiO_2_), to enhance the mechanical properties and wettability of green electronic devices [19]. Among them, Al is widely used as an alloying element for refining the microstructure, and improving the solderability, and the mechanical properties [20]. Das et al. reported that the melting temperature of Sn-9Zn eutectic solder alloy decreased slightly with the addition of Al, and that the micro-hardness value and tensile strength of Sn-9Zn-0.5Al was higher compared to the Sn-9Zn eutectic solder alloy [21]. In addition, wettability and ductility were improved with the addition of Al to Sn-9%Zn solder because of the decrease in oxygen concentration at the solder surface. The strength of the boundaries concentrating Al element was without any loss [22]. Li et al. reported that additions of 1–2% of Al into the basic Sn-58Bi solder alloy appeared to accumulate and oxidize at the surface of solder at both 200 °C and 240 °C. This prevented the Al atoms from reaching the solder/Cu interface to form the type of Cu-Al IMC [23]. Li et al. discovered that the coarsening rate of rich-Bi phase declined and the trend of IMC growth slowed down at the solder joint with the addition of Al, but the spreading property of the alloy decreased due to Al existing in alloy by Al particle and Al atom solid solution [24]. Furthermore, the Sn-Al solder alloy has been investigated. Due to the combination effect of Al, the strength of the Sn-Al alloy was improved and the plasticity was decreased compared with that of pure Sn [25]. Shnawah et al. discovered that the addition of 0.1 wt.% Al formed coarser IMC particles than the addition of 0.5% Al [26].

Wettability and mechanical properties are of fundamental importance to solder joints. Until now, studies on non-eutectic Sn-20Bi solder alloys with Al have not been reported. The cost of Al is relatively low. Based on the effects of Al addition in Sn alloys, as reviewed above, this study selected Al as the alloying element to improve the wettability and mechanical properties, and to modify the microstructure, of Sn-20Bi alloys. Therefore, in this study, we investigated the effects of Al concentration on the microstructure, wettability and mechanical properties of the Sn-20Bi solder alloy to produce a viable solder alloy.

## 2. Experimental Procedure

Bismuth powder (purity 99.95 wt.%, −200 mesh), Tin powder (purity 99.99 wt.%, −200 mesh), and Aluminum powder (purity 99.9 wt.%, −200 mesh) were used in this study as raw materials. A planetary ball mill (BXQM-2L, Nanjing, China) was used for the MA of power. The powder mixture in the stoichiometric ratio (Sn-20Bi, Sn-20Bi-0.01Al, Sn-20Bi-0.1Al, Sn-20Bi-0.3Al, Sn-20Bi-0.5Al) was sealed under an argon atmosphere. The ball-to-powder weight ratio was maintained at around 15:1. In all the experiments, ratio speed was fixed at 150 rpm and total time was 180 min. Then the mixture alloy powder was compacted uniaxially (close die) to attain a tight alloy piece. Compaction pressure was 80 MPa and die diameter was Φ20 mm. Finally, the alloy samples were produced by arc melting on a water-cooled Cu cast with a non-consumable tungsten electrode in a pure argon atmosphere. Homogeneous Sn-20Bi-xAl (x = 0 wt.%, 0.1 wt.%, 0.3 wt.%, 0.5 wt.%) alloys were obtained. The water for the experiment was obtained from Milli-Q Advantage A10.

### 2.1. Microstructural Observation

Specimens were mounted in epoxy. The mounted specimens were grinded and polished according to standard metallographic techniques. The microstructure was observed using the scanning electron microscope (SEM, Hitachi S-3400, Tokyo, Japan) with a voltage of 20 keV. Energy dispersive spectroscopy (EDS) microanalysis was also used to determine the elemental composition at the selected areas of solder alloy. The phases of the alloy samples were identified using an X-ray diffraction (XRD, Rigaku D/Max 2500V, Tokyo, Japan) using CuK_α_ radiation, operating at 40 KV, 200 mA and a scanning rate of 8 (°)/min at diffraction angle 2θ from 20° to 90°.

### 2.2. Micro-Hardness Test

The polished samples were placed in a Vickers micro-hardness tester (HVT-1000, Shanghai, China) with a pyramid-shaped diamond to measure the micro-hardness. The applied load was 30 g for 15 s, and at least 5 readings of different indentations were taken at room temperature to obtain the mean value.

The micro-hardness of a material is often related to its wear behavior and determines the durability of a material during its various applications. Furthermore, it is a micro-hardness measurement technique that is used to determine the hardness of total grains, phases, and structural components of a material. The value of micro-hardness of a material depends on the grain size, motion of dislocations, the growth, and the configuration of grains [27].

### 2.3. Spreading Test

In order to prepare the specimens for the spreading test, the circle plates were cut from Sn-20Bi-xAl solder alloys with 6.5 mm diameter and 1.24 length. The spreading area and the contact angle on the copper substrates were measured using a wetting balance in accordance with the JISZ3198 standard. Copper plates of 40 mm × 40 mm × 2 mm were grinded. Acetone was used to eliminate surface defects such as oil and blots, followed by washing in distilled 10% HCl solution to remove surface oxide and contaminates. Then the copper substrates immersed in 3% NaOH solution and cleaned in deionized water. Thereafter, prepared circle pieces were placed on Cu plates to be manually soldered at the temperature of 250 °C. After soldering, top-view and cross-view optical images of Sn-Bi-based solder alloys spreading on substrates can be acquired by Axio Imager 2 (Carl Zeiss, Jena, Germany).

### 2.4. Potentiodynamic Polarization Measurement

Electrochemical measurements were carried out in a single compartment cell, using a standard three-electrode configuration: saturated calomel electrode (SCE) as a reference, with a platinum electrode as counter, and a sample as the working electrode. The surface area exposed to the test solution was 1 cm^2^. The specimens were given a metallographic polishing prior to each experiment, followed by washing with distilled water and acetone. All the experiments were performed in aerated 3.5 wt.% NaCl solution at room temperature (25 ± 0.1 °C). Potentiodynamic polarization curves were recorded in the potential range −2000 to 0 mV vs. SCE reference electrode at a scan rate of 2 mV/s, after allowing a steady-state potential to develop.

## 3. Results and Discussion

### 3.1. Microstructure of Sn-Bi-Al Solder Alloy

**Figure 1:**
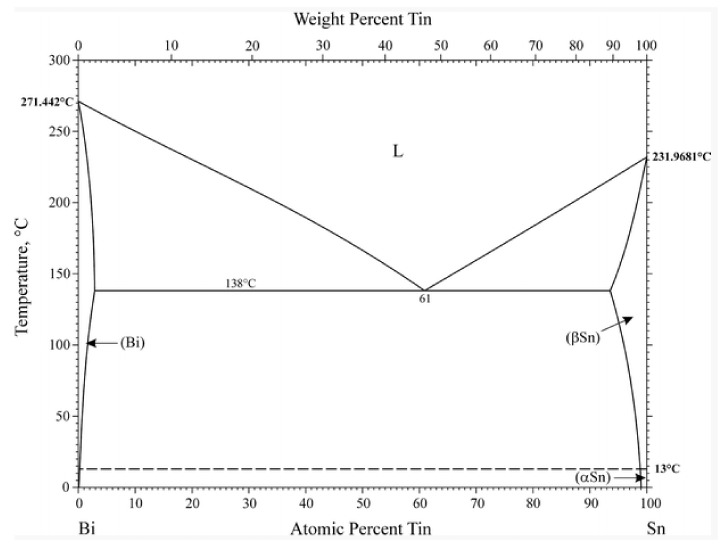

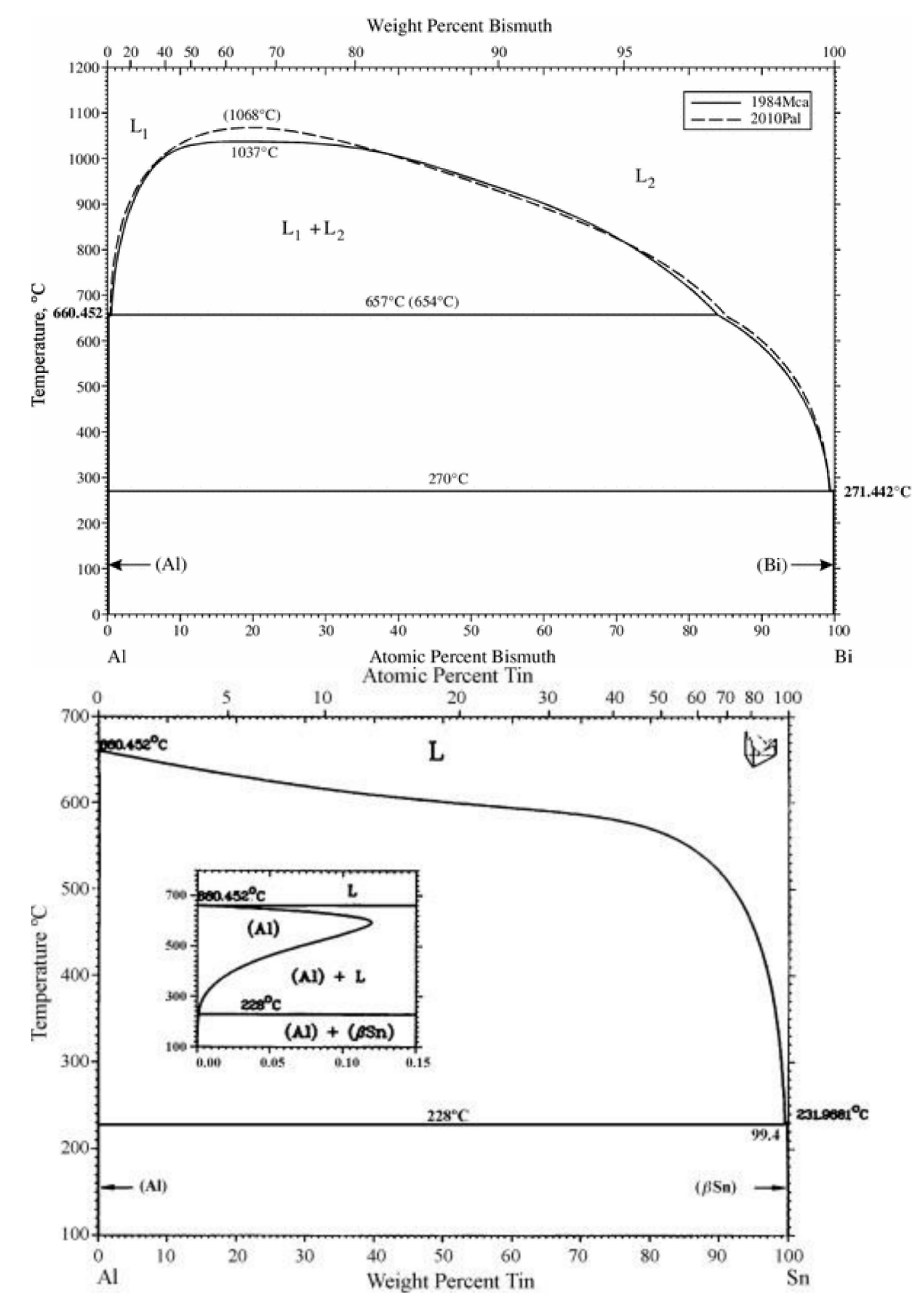
Bi-Sn, Al-Bi, Al-Sn phase diagrams [28].

**Figure 2:**
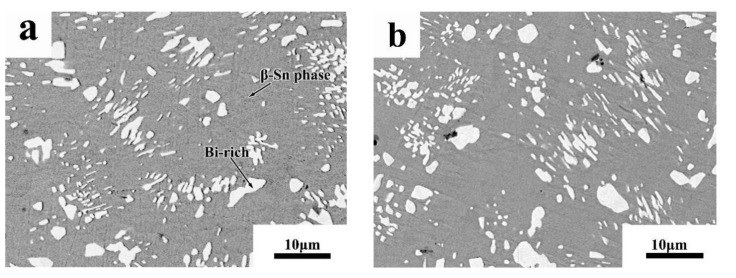

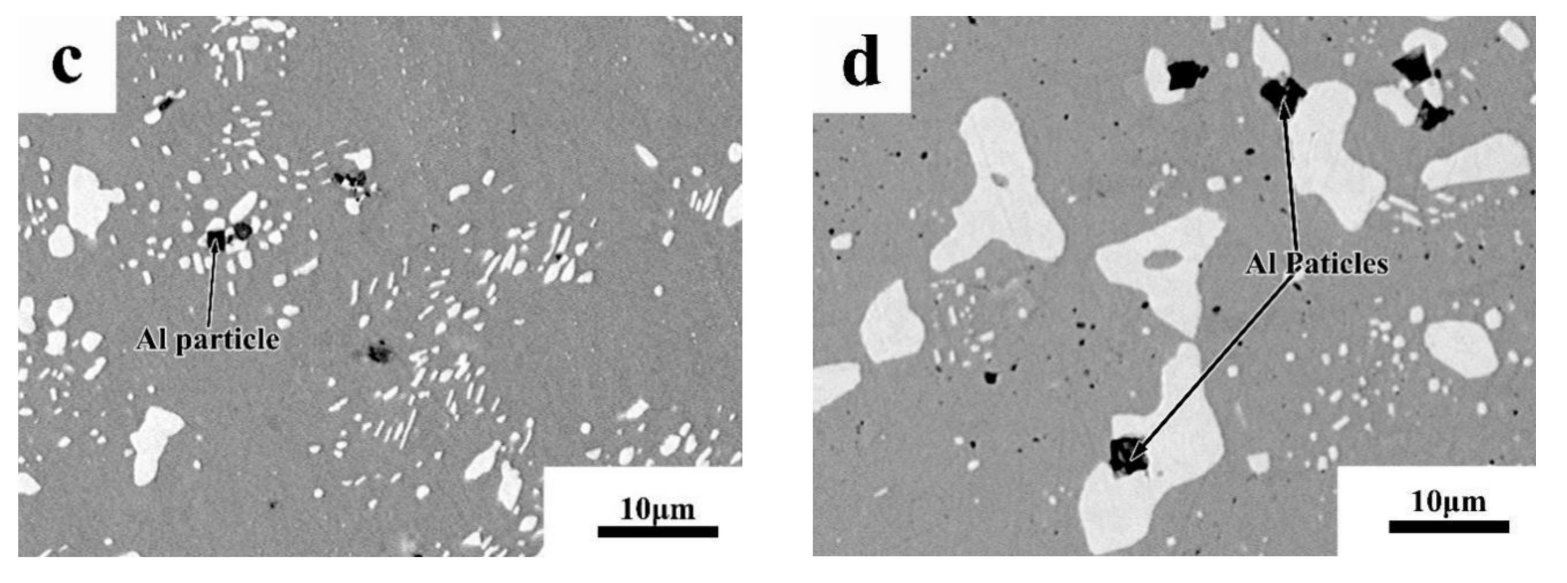
High-magnification SEM microstructures of Sn-20Bi-xAl Solder alloys: (**a**) Sn-20Bi, (**b**) Sn-20Bi-0.1Al, (**c**) Sn-20Bi-0.3Al, (**d**) Sn-20Bi-0.5Al.

**Figure 3:**
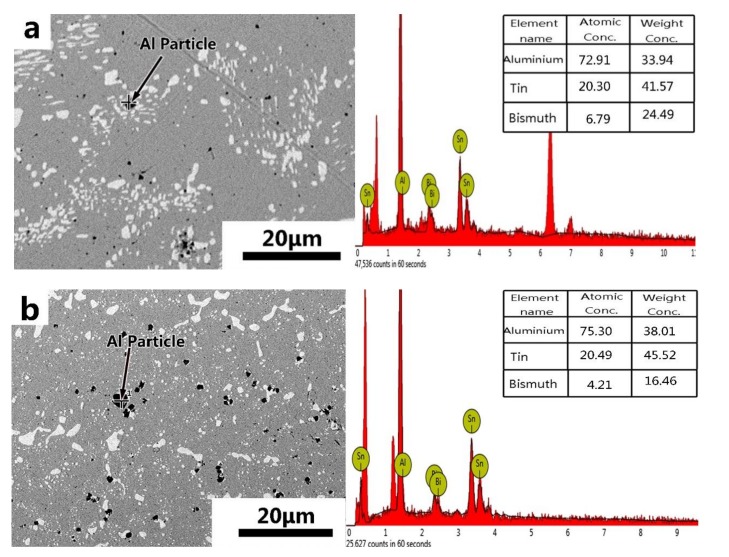
Energy dispersive spectroscopy (EDS) results of the third phase in (**a**) Sn-20Bi-0.3Al and (**b**) Sn-20Bi-0.5Al solder alloy.

**Figure 4:**
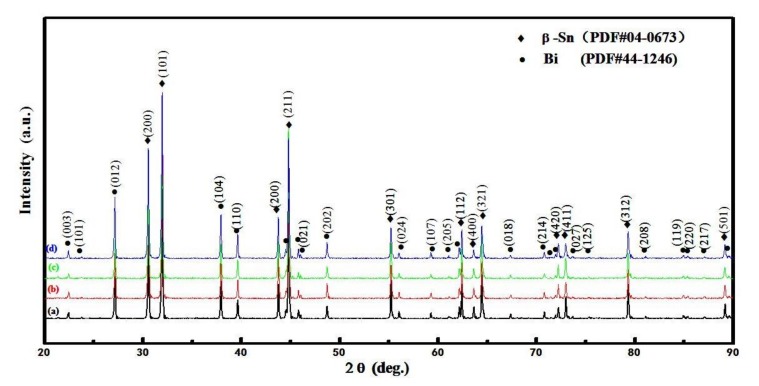
X-rays diffraction pattern for (**a**) Sn-20Bi, (**b**) Sn-20Bi-0.1Al, (**c**) Sn-20Bi-0.3Al, (**d**) Sn-20Bi-0.5Al Solder alloys.

The phase diagrams of Bi-Sn, Al-Bi, Al-Sn are shown in Figure 1. Scanning electron microscopy (SEM) images in Figure 2 and Energy dispersive spectroscopy (EDS) in Figure 3 reveal the variation of the microstructures in samples with different Al concentrations. The result of the XRD shown in Figure 4 reveals that it consists of primary β-Sn and Bi phases. In Figure 2, the dark regions are rich-Sn phases and the primarily solidified phases, the bright regions are fine Bi-rich particles and are uniformly distributed in the β-Sn matrix. The microstructure gradually coarsened with the increase of Al in Figure 2. Reference [11] also reported that particle-shaped Bi phases were found distributed in Sn-rich phase matrix. Referring to the equilibrium Sn-Bi phase diagram [28], the reaction is L(liquid)→L + primary Sn →primary Sn + Bi. A third phase was found in Figure 2c,d. It is a regular polygonal shape. The black regular phase composition in Sn-20Bi-0.3Al and Sn-20Bi-0.5Al was analyzed by energy dispersive spectroscopy (EDS). The results are shown in Figure 3. The Black regular phase is particle shape Al-rich. In the Sn-20Bi-xAl Solder alloys, Al did not react with Bi and Sn. Alam et al. also discovered that Al atom in Sn-Al solder alloy existed as Al particle [25]. In Figure 2, the sizes of the Bi-rich particles increased as the Al fraction increased. When Al fraction increased to 0.5 wt.%, the Bi-rich phase formed a lightly segregated pattern. Al is known as a grain refiner which can promote nucleation. The addition of Al could thus enhance the nucleation and growth of the Bi-rich phase. Thus, it was concluded that the morphology of the Bi-rich phase did not have obvious variation when the fraction of Al was less than 0.3 wt.%. However, when the content of Al was 0.5 wt.%, the Bi-rich phases become coarser.

### 3.2. Micro-Hardness Analysis

**Figure 5:**
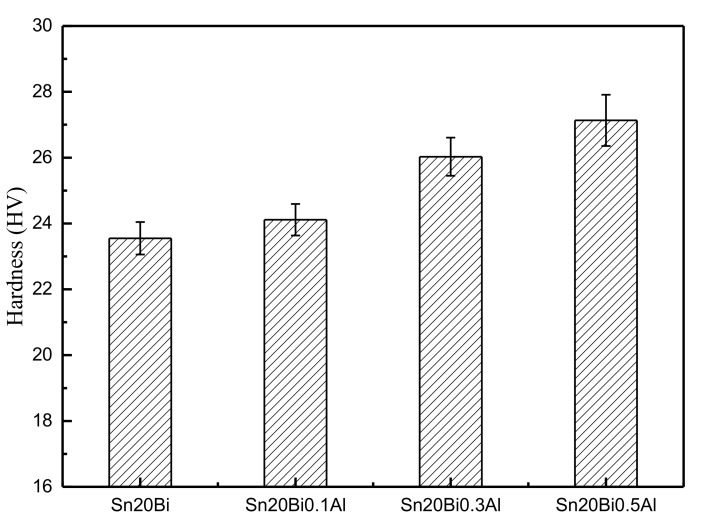
Micro-hardness of Sn-20Bi-xAl solder alloy.

The results obtained from the micro-hardness tests of Sn-20Bi-xAl alloys are shown in Figure 5. The experimental results are an average of five indentations at different points on the solder alloy surface.

The hardness of Sn-20Bi-xAl improved with the increasing of Al fraction. When the fraction of Al was 0.5 wt.%, the micro-hardness was the highest by 27.1 HV and increased by 15.2%, compared to the microhardness of Sn-20Bi at 23.6 HV. The hardness change of alloy is slight compared to Sn-20Bi alloy when the fraction of Al is 0.1 wt.%. Sn-20Bi-0.1Al slightly decreases by 2.4%. Wang et al. suggested the dispersion strengthening mechanism, which distributed the hard particles within the β-Sn matrix, act as the pining centers for the movement of dislocation [29]. Thus, the increase in micro-hardness of the solders containing Al elements is related to the particle strengthening. A homogeneous distribution of Al particles in the solder matrix can give rise to better dispersion strengthening, resulting in an increase in micro-hardness of Sn-20Bi-xAl alloy. However, when the fraction of Al is 0.5 wt.%, the Bi-rich phase generated segregation and coarsened, which is mentioned in the preceding part of this text. So, the change value of hardness gradually became flat when the content of Al was more than 0.3 wt.%.

### 3.3. Wettability

**Figure 6:**
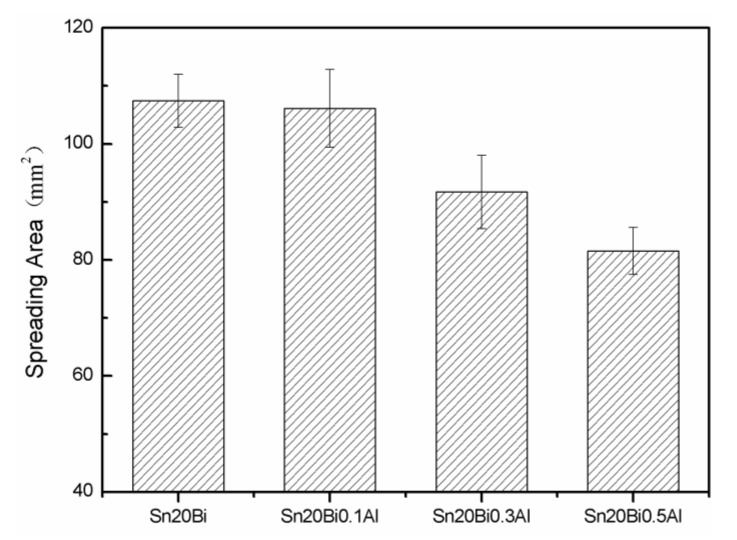
Spreading area of Sn-20Bi-xAl solder alloy.

**Figure 7:**
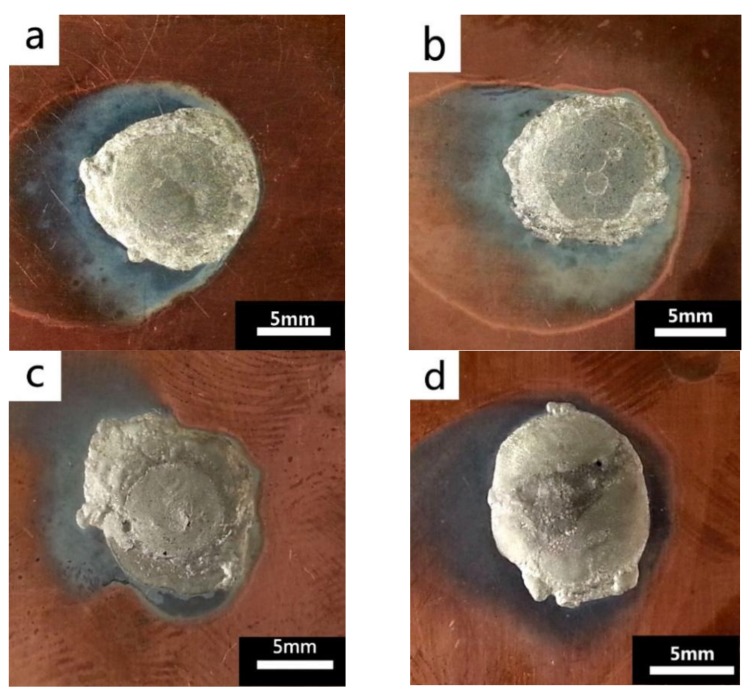
Top-view optical images of Sn-Bi-based solder alloys spreading on substrates: (**a**) Sn-20Bi, (**b**) Sn-20Bi-0.1Al, (**c**) Sn-20Bi-0.3Al, and (**d**) Sn-20Bi-0.5Al.

The wettability of solder alloy plays an important role in reliable joins. The spreading area and wetting angle was measured to evaluate the wettability for the molten solder [18]. The top-view optical images of Sn-Bi-based alloy molted on pure copper were processed by AutoCAD software and spreading areas were acquired [30]. The data points are the average of five measurements on each sample. Figure 6 shows the spreading area of Sn-20Bi-xAl solder alloys on Cu substrate. And the top-view optical images of Sn-Bi-based solder alloys spreading on substrates are shown in Figure 7. With an increase in Al content of the solder, the spreading area decreased continuously. For Sn-20Bi-0.1Al, the spreading area was 106.1 mm^2^ and only decreased by 1.2% compared to the spreading area of Sn-20Bi. When the content of Al increased to 0.3 wt.% and 0.5 wt.%, the spreading area decreased by 14.6% and 24.1%, respectively. It can be concluded that the wettability of Sn-20Bi-0.1Al solder alloy had a slight reduction related to Sn-20Bi-0.3Al and Sn-20Bi-0.5Al alloys.

The extent of wetting is expressed by γ_sf_, which is calculated from the following equation [31]:

γ_sf_ = γ_sl_ + γ_lf_cosθ (1)

where it is related to the contact angle(θ), the interfacial tensions of Substrate/flux(γ_sf_), liquid-solder/flux(γ_lf_), and substrate/liquid solder(γ_sl_) interface. When the value of the contact angle(θ) is lower, the wetting ability is better [32]. Triplicate experimentation of the contact angle was measured for each sample. Table 1 shows cross-view optical images and the angle was measured by Axio Vision rel.4.8 software. For the Sn-20Bi, Sn-20Bi-0.1Al, Sn-20Bi-0.3Al, and Sn-20Bi-0.5Al solder alloy, the means of the contact angle were 14.49°, 17.32°, 20.92°, and 26.04°, respectively. When the content of Al increased, the wetting angle increased. According to the Young–Dupree equation, a small wetting angle requires a large (γ_sf_ − γ_sl_) or a small γ_lf_ value. When the content of Al was tiny, Al was soluble in the Sn matrix [25]. Thus, the wetting angle was slightly influenced by the trend of the spreading area. As 0.3 wt.% and 0.5 wt.% Al was added, redundant Al atoms existed in free state. When solder alloy was melted in 250 °C, Al atoms in free state were still solid and the film was formed on the surface of the molten solder. In addition, the Al atom was active and could of been easily oxidized. During the spreading process, Al could of formed an oxide layer. Hence, a higher γ_lf_ value was achieved and the wetting ability was dropped. Therefore, γ_lf_ became larger with the addition of Al, which resulted in the larger θ, thus the wetting ability is dropped. When the weight ratio of Al is 0.1%, there was no dramatic change in the wetting ability.

### 3.4. Corrosion Resistance

**Figure 8:**
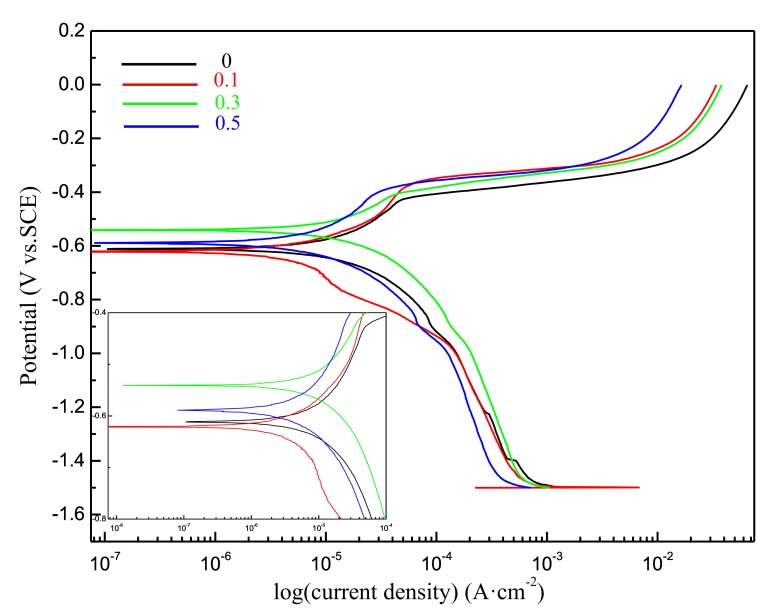
Potentiodynamic polarization curves of Sn-20Bi-xAl (x = 0, 0.1, 0.3, 0.5) solder alloys.

The polarization studies provide interpretation of the corrosion behavior of the corresponding solder alloys. Figure 8 represents typical polarization curves (log I = f(E)) obtained for Sn-20Bi-xAl (x = 0, 0.1, 0.3, 0.5) solder alloys in naturally aerated 3.5 wt.% NaCl solution. The electrochemical corrosion parameters are listed in Table 2, which shows the effect of the Al content on the electrochemical parameters of the different solders. The corrosion rate of Sn-20Bi-0.3Al was the biggest. Adding 0.3 wt.% Al to Sn-20Bi solder alloy increased the corrosion current. Because the Al-rich phase is formed in the Sn matrix when the content of Al is more than 0.3 wt.%, it destroyed the continuity of the matrix. In addition, the standard electrode potential of Al is −1.662 V was lower than Sn and Bi. Therefore, Al would take the priority if the dissolution reaction was attacked. The reactions occurred according to the following steps [33]:

Al + 3OH^−^ − 3e^−^ → Al(OH)_3_ (2)

The corrosion products became greater with the increase of Al and formed enough of a Al(OH)_3_ layer to prevent further corrosion. We can also see that the 0.5 wt.% Al solder had a much lower I_c_ value of 3.91 × 10^−6^ A/cm^2^ than that of the 0.3 wt.% Al solder. Adding 0.1 wt.% Al to Sn-20Bi solder alloy improved the corrosion resistance and decreased the corrosion current to 3.49 × 10^−6^ A/cm^2^. Minor Al was dissolved in β-Sn matrix. The dissolution of Sn was the only dissolution for solder alloys. Sn dissolution occurs according to the following reaction [34]:

3Sn + 2Cl^−^ + 4OH^−^ − 6e^−^ → Sn_3_O(OH)_2_Cl_2_ + H_2_O (3)

All of the above results suggest that Sn-20Bi-0.1Al has the best corrosion resistance, and the reliability of the solder is expected to improve to a certain extent, which might be useful to the electronics industry.

**Figure 9:**
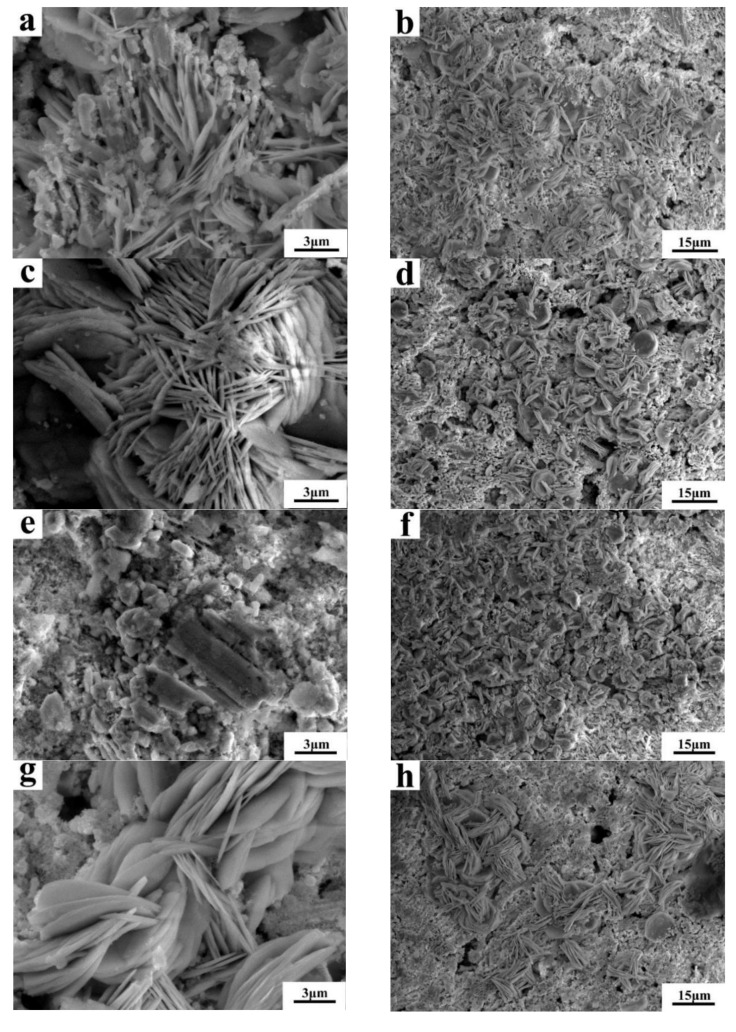
SEM of Sn-20Bi-xAl after polarized to 0 V in 3.5 wt.% NaCl solution: (**a**,**b**) Sn-20Bi, (**c**,**d**) Sn-20Bi-0.1Al, (**e**,**f**) Sn-20Bi-0.3Al, and (**g**,**h**) Sn-20Bi-0.5Al.

Figure 9 shows the SEM of Sn-20Bi-xAl (x = 0, 0.1, 0.3, 0.5) solder alloys after polarized to 0 V in 3.5 wt.% NaCl solution. Figure 9a,c,e,g are respectively the high magnification figures, and Figure 9b,d,f,h are the low magnification figures, of Sn-20Bi-xAl solder alloys. From the micrograph, the corrosion products of alloys are Sn_3_O(OH)_2_Cl_2_. It can be observed that the solder alloys corrosion products exhibit lamerllar-like structures. The pit appears in Figure 9e which is caused by the exfoliation of the Sn-20Bi-0.3Al alloy corrosion products. It has been mentioned above that the corrosion of Sn-20Bi-0.3Al alloy is the most serious. Comparing Figure 9a,c, it can be seen that the corrosion product of the alloy with 0.1 wt.% Al added is more dense, so the corrosion resistance of Sn-20Bi-0.1Al alloy is the best.

## 4. Conclusions

The microstructure, hardness, and wettability of Sn-20Bi-xAl solder alloys were investigated and following conclusions were derived from this study:

(1) The microstructure of Sn-20Bi-xAl solder alloys changed as the content of Al increased, and consisted of primarily β-Sn and particle-shaped Bi phases. Al particles appeared in Sn-20Bi-0.3Al and Sn-20Bi-0.5Al, which is of black and regular morphology. When the Al fraction increased to 0.5 wt.%, the Bi-rich phase formed a light segregation pattern and became coarser, while the morphology of the other alloys did not obviously change.
(2) The hardness of Sn-20Bi-xAl improved increasingly with the increase of the Al fraction. Hardness increased to a maximum of about 27.1 HV when the fraction of Al is 0.5 wt.%. When the content of Al was more than 0.3 wt.%, the change value of hardness gradually became flat.
(3) The spreading area decreased gradually in the Sn-20Bi-xAl alloys when Al was added. The contact angles θ had a monotone increase with the increase of Al. The contact angles are 14.49°, 17.32°, 20.92°, and 26.04 °, respectively. The Al fraction effected the wettability slightly when the Al fraction was less than 0.1 wt.%. However, Sn-20Bi-0.3Al and Sn-20Bi-0.5Al solder alloys dropped sharply in wettability.
(4) The corrosion products of Sn-20Bi-xAl alloy are lamerllar-like Sn_3_O(OH)_2_Cl_2_. The corrosion resistance of Sn-20Bi-0.1Al solder alloy is the best, when the corrosion current is the lowest at 3.49 × 10^−6^ A/cm^2^ due to the dense corrosion products. However, when 0.3 wt.% was added to the Sn-20Bi alloy, the solder alloy was easiest to corrode and a pit was caused by the exfoliation of the corrosion products.

## Figures and Tables

**Table 1:** Cross-view optical images of Sn-Bi-based solder alloys spreading on substrates.

Alloys	1st	2nd	3rd	Mean (°)
Sn-20Bi	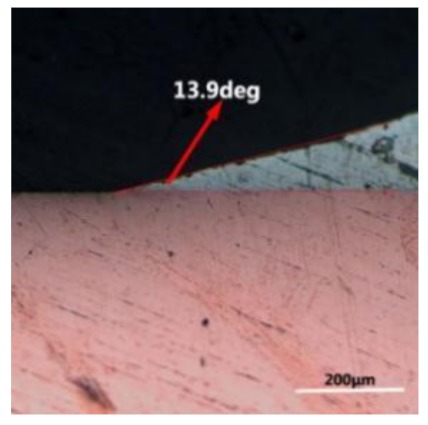	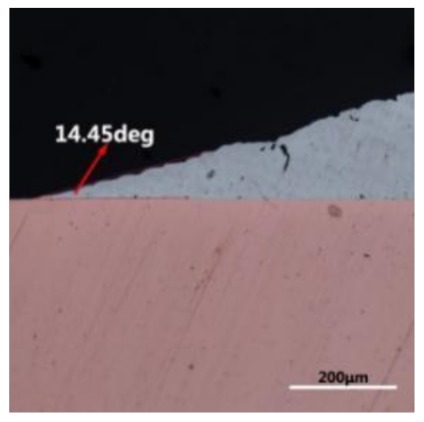	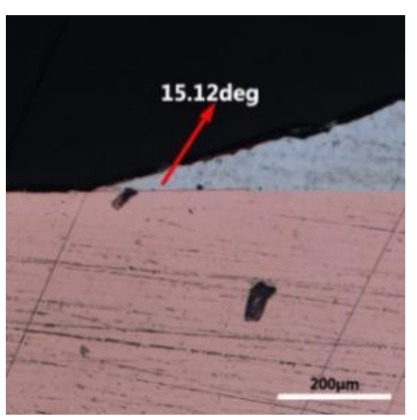	14.49
Sn-20Bi-0.1Al	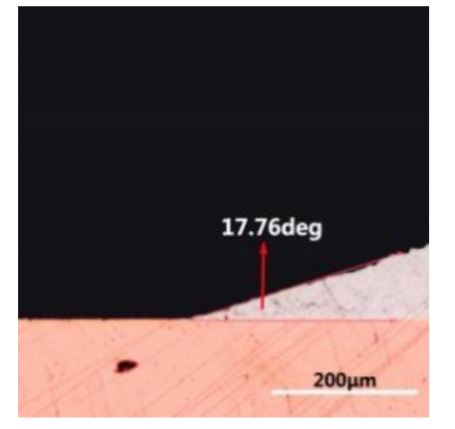	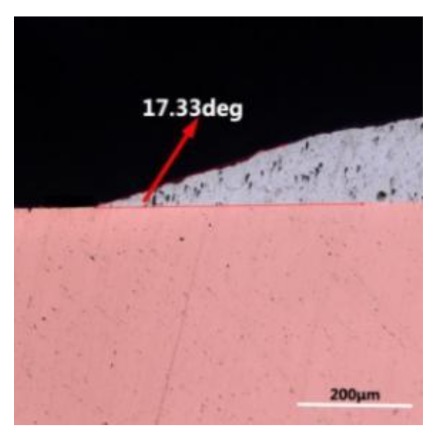	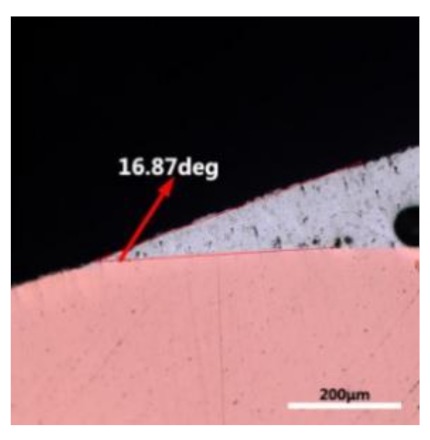	17.32
Sn-20Bi-0.3Al	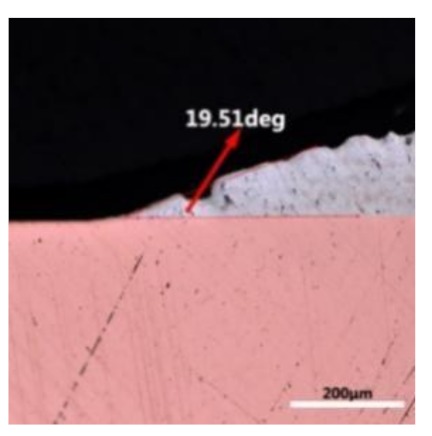	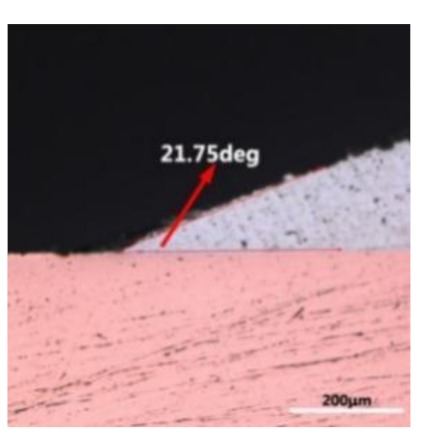	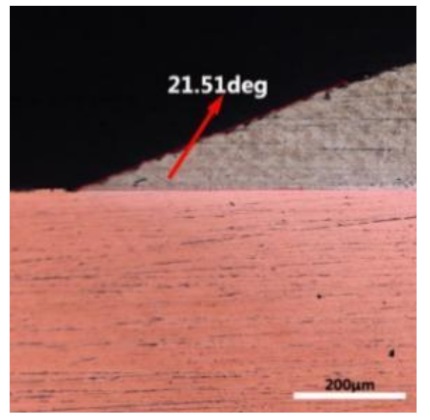	20.92
Sn-20Bi-0.5Al	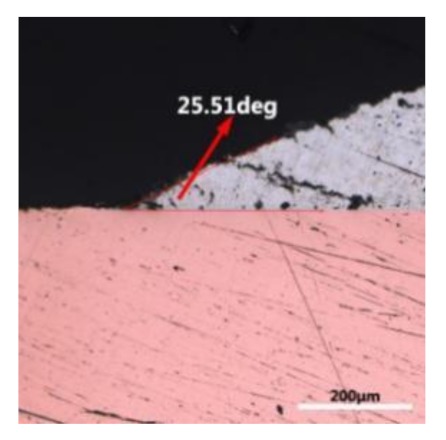	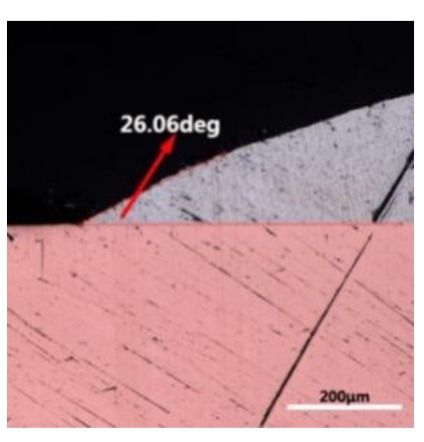	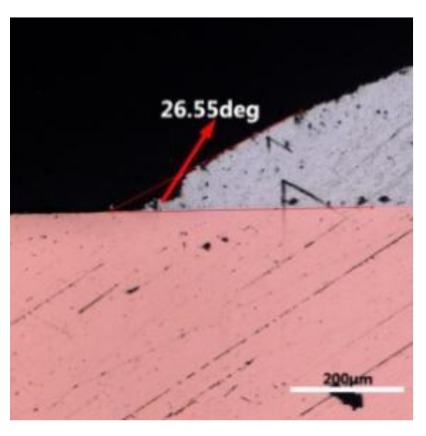	26.04

**Table 2:** The related parameters of Sn-20Bi-xAl alloys after polarized in 3.5 wt.% NaCl solution.

Alloy	I_corr_ (A/cm^2^)	E_corr_ (V)	Corrision Rate (mm/a)
Sn-20Bi	5.74 × 10^−6^	−0.61405	0.15215
Sn-20Bi-0.1Al	3.49 × 10^−6^	−0.62950	0.09259
Sn-20Bi-0.3Al	8.20 × 10^−6^	−0.53693	0.21720
Sn-20Bi-0.5Al	3.91 × 10^−6^	−0.59243	0.10371
